# Detection of chromosomal and gene abnormality with karyotyping, chromosomal microarray analysis and trio-based whole exome sequencing in pregnancies with fetal growth restriction: implications for precise prenatal diagnosis

**DOI:** 10.1186/s12884-025-08164-0

**Published:** 2025-10-10

**Authors:** Yangping Chen, Meiying Cai, Meihuan Chen, Xiaohe Pan, Xuna Shen, Binbin Song, Xiaohe Cai, Lili Zhou, Hailong Huang

**Affiliations:** 1 College of Clinical Medicine for Obstetrics & Gynecology and Pediatrics, Fujian Medical University, Fuzhou, Fujian Province 350004 China; 2 Department of Obstetrics and Gynecology, Wenzhou Central Hospital, Wenzhou, 325000 Zhejiang Province China; 3 Medical Genetic Diagnosis and Therapy Center of Fujian Maternity and Child Health Hospital, Fujian Provincial Key Laboratory of Prenatal Diagnosis and Birth defects, No. 18 Daoshan Road, Fuzhou, Fujian Province 350001 China; 4 Center for Prenatal Diagnosis, Wenzhou Central Hospital, Wenzhou, 325000 Zhejiang Province China

**Keywords:** Fetal growth restriction, Karyotyping, Chromosomal microarray analysis, Trio-based whole exome sequencing, Prenatal diagnosis

## Abstract

**Background:**

Although extensively investigated, the genetic etiology of fetal growth restriction (FGR) has not been fully understood. Previous studies have shown the potential of karyotyping, chromosomal microarray analysis (CMA) and trio-based WES (trio-WES) in prenatal diagnosis, however, there is little knowledge on the comparative effectiveness of these three prenatal genetic tools. The present study aimed to evaluate and compare the effectiveness of karyotyping, CMA and trio-WES for prenatal diagnosis and prognostic evaluation of FGR, in order to identify the optimal genetic diagnostic testing of FGR.

**Methods:**

All clinical data were retrospectively collected from 388 pregnant women that received invasive prenatal diagnosis due to FGR in Wenzhou Central Hospital and Fujian Maternity and Child Health Hospital during the period from April 2015 to August 2024. All fetuses received karyotyping and CMA, and additional trio-WES was performed among 26 pregnancies negative for karyotyping and CMA. The effectiveness of karyotyping, CMA, and trio-WES alone and in combinations was evaluated for diagnosis of chromosomal abnormality in pregnancies with FGR, and the pregnancy outcomes were followed up.

**Results:**

Karyotyping detected a 4.38% (17/388) chromosomal abnormality rate and CMA detected a 13.4% (52/388) chromosomal abnormality rate, which resulted in a 9.28% (36/388)incremental yield above karyotyping. The diagnostic yield of Karyotyping combined with CMA was 13.66% (53/388). The common microdeletion and microduplication syndromes included 4p16.3.3 microdeletion syndrome, 1q21.2 microduplication syndrome, 16p13.3 microdeletion syndrome and 22q11.21 microduplication syndrome. Trio-WES revealed a high detection rate (30.77%,8/26) for diagnosis of P/LP variants, and the growth- and development-related genes included *IGF1R*, *GNAS*, *COL9A3*, *FGD1*, *FLNB*, *PORCN*, *EVC*, *OTX2* and *GH1*. Of all pregnancies with FGR, the proportions of live births, induced labors, natural abortion, perinatal mortality, and percentage of loss to follow-up were 70.62% (274/388), 19.84% (77/388), 0.52% (2/388), 1.80% (7/388) and 7.22% (28/388), respectively.

**Conclusions:**

Karyotyping combined with CMA remains the first-tier tool for prenatal genetic diagnosis of pregnancies with FGR, and additional trio-WES may increase the detection of chromosomal abnormality. *IGF1R*, *GNAS*, *COL9A3* and *FGD1* may be candidate genes causing FGR.

## Introduction

Fetal growth restriction (FGR), a common pregnancy complication, is defined as a fetal failure to reach its genetically predetermined growth potential, with estimated fetal weight (EFW) or abdominal circumference (AC) less than the 10th percentile for gestational age, as revealed by ultrasound evaluation [1]. Currently, FGR is estimated to affect 5–10% of total pregnancies and has been identified as a major determinant of perinatal morbidity and mortality [2]. More importantly, fetuses experiencing FGR are at an increased risk of physical development retardation, intellectual developmental disorder, and learning and cognitive deficits during childhood and adolescence, and are more Likely to develop obesity, type 2 diabetes mellitus, cardiovascular diseases and stroke in adults [3]. Nevertheless, there is currently no effective cure for FGR, and there is no evidence proving that maternal oxygen administration during pregnancy, fetal protection or nutrients supplementation can improve FGR [4].

The pathogenesis of FGR has been extensively investigated, and multiple etiologies have been proposed, including fetal genetic abnormalities, placental transport factors and insufficient maternal nutritional supply, with fetal genetic abnormalities contributing 15–20% to the pathogenesis of FGR [5]. A recent systematic review and meta-analysis showed a 6.4% pooled proportion of chromosomal aberrations in pregnancies with apparently isolated FGR [6], and chromosomal microarray analysis (CMA) was reported to increase the detection rate of isolated FGR by 4.4% over karyotyping [7]. A recent study reported that karyotyping analysis detected a 28.5% proportion of chromosomal abnormalities among pregnancies with FGR accompanied by structural malformations, and CMA revealed 7.7% additional detection rate over karyotyping [8]. In addition, results from a systematic review and meta-analysis showed that WES detected an additional diagnostic yield rate of 33% over negative karyotyping and CMA results among multisystem fetal structural anomalies [9].

The American College of Obstetricians and Gynecologists (ACOG) Practice Bulletin on Fetal Growth Restriction released by the ACOG and the Society for Maternal-Fetal Medicine in 2021 recommended genetic counseling and prenatal diagnosis given for pregnant women with FGR combined with polyhydramnios, fetal malformation, or diagnosis of FGR prior to 32 weeks of gestation [10], however, there is no consensus on the application of WES in diagnosis of FGR. If FGR is a result of congenital genetic material abnormalities, the poor outcomes of perinatal morbidity or mortality cannot be improved either in uterine or after birth [11]. Precise prenatal diagnosis of genetic etiology is therefore of great importance for the clinical management of FGR [12]. The present study aimed to investigate the genetic etiology of 388 pregnancies with FGR, and evaluate and compare the effectiveness of karyotyping, CMA and prenatal trio-WES for prenatal diagnosis and prognostic evaluation of FGR, in order to identify the optimal genetic diagnostic testing of FGR.

## Subjects and methods

### Subjects

**Fig. 1:**
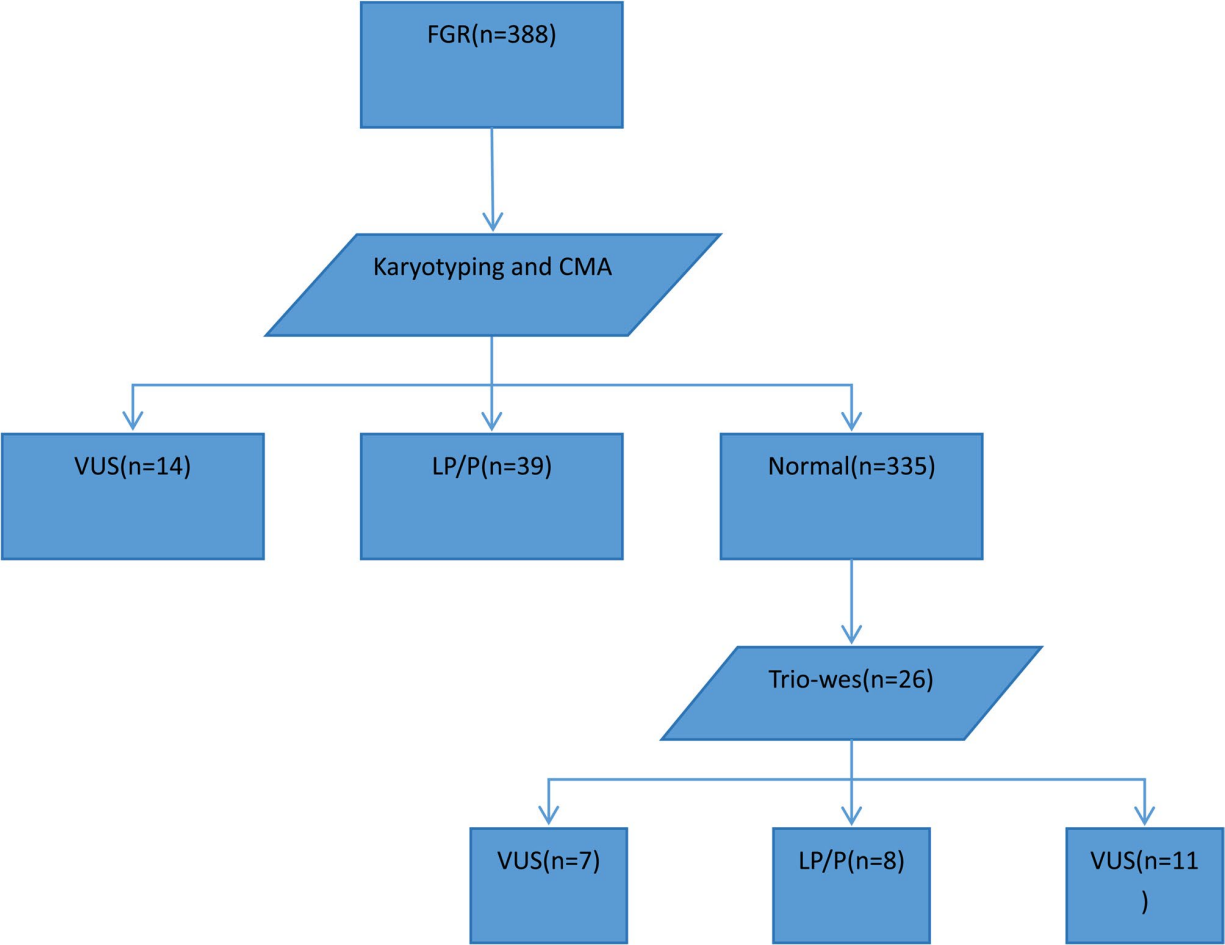
Flowchart for detection of chromosomal and gene abnormality in pregnancies with fetal growth restriction using karyotyping, chromosomal microarray analysis and trio-based whole exome sequencing

The demographic and clinical characteristics were captured from 388 pregnant women who received invasive prenatal diagnosis due to FGR in Wenzhou Central Hospital (Wenzhou, China) and Fujian Maternity and Child Health Hospital (Fuzhou, China) during the period from April 2015 to August 2024. The pregnant women were aged between 16 and 45 years and had gestational ages of 18 to 32weeks. If a pregnant woman at a gestational age of over 32 weeks would Like to undergo trio-WES, they will be informed of a high risk of delivery before trio-WES results are acquired. In this study, the largest gestational age of pregnant women participating in trio-WES is 31 weeks and 3 days. Singleton pregnancy with EFW or AC less than the 10th percentile, or less than two standard deviations below the mean (M-2SD) for gestational age by ultrasound evaluation was included, and FGR caused by maternal factors, such as long-term malnutrition, gestational hypertension, placental dysfunction or cytomegalovirus infections, was excluded. All participants were classified into the isolated FGR group (*n* = 227), FGR complicated by a single ultrasound soft marker group (*n* = 76), FGR complicated by multiple ultrasound soft markers group (*n* = 52) and FGR complicated by ultrasound structural abnormality group (*n* = 33). Amniocentesis was performed at gestational ages of 18 to 26 weeks, and cordocentesis was performed after 26 weeks of gestational age. (Fig. 1)

### Karyotyping analysis

Approximately 20 mL of amniotic fluid samples were collected during amniocentesis, and transferred to two sterile EP tubes, and umbilical cord venous blood samples were collected and transferred to two blood collection tubes containing heparin and ethylenediaminetetraacetic acid (EDTA) anticoagulants, respectively. Amniotic fluid or umbilical cord venous blood samples were routinely processed for chromosome G-banding karyotype analysis [13], and the karyotyping results were interpreted using the International System for Human Cytogenomic Nomenclature [14].

### CMA analysis

CMA analysis was performed with the 12-sample HumanCytoSNP-12 BeadChip (Illumina, Inc.; San Diego, CA, USA) as described previously [15]. All original data were transformed with the KaryoStudio and GenomeStudio software, and copy number variations (CNVs) were analyzed and aligned with the Human Database of Genomic Variants (http://dgv.tcag.ca/dgv/app/home) to identify polymorphic CNVs. The variant pathogenicity was analyzed using the databases DECIPHER, Database of Genomic Variants (DGV), Online Mendelian Inheritance in Man (OMIM), UCSC Genome Browser, ENSEMBL and Chinese Chromosome Health Database, and CNVs were classified into benign, likely benign, variants of uncertain clinical significance (VUS), likely pathogenic (LP) and pathogenic (P), according to the 2019 American College of Medical Genetics and Genomics (ACMG) guideline for classification of CNVs [16]. Several laboratory-based assays, including multiplex ligation-dependent probe amplification (MLPA), fluorescence in situ hybridization (FISH), quantitative fluorescent polymerase chain reaction (qPCR) assay and RNA sequencing, were employed to evaluate the accuracy of karyotyping and single nucleotide polymorphisms (SNPs) microarrays for detection of LP CNVs. VUS smaller than 500 kb deletion or 1 Mb duplication, VUS larger than 500 kb deletion or 1 Mb duplication with insufficient or conflicting evidence supporting disease association, polymorphic CNVs suggested by public databases, loss of heterozygosity (LOH) smaller than 10 Mb, or absence of heterozygosity (AOH) involving sex chromosome were not reported.

### WES

If karyotyping and CMA were tested negative, prenatal WES would be performed to detect the exon region and neighboring intron regions of 7,099 genes in human exome on a NextSeq 550Dx system (Illumina, Inc.), and all variants of established clinical significance were validated using Sanger sequencing. The pathogenicity of variants was interpreted following the guidelines for the interpretation of sequence variants recommended by the American College of Medical Genetics and Genomics and the Association for Molecular Pathology [17], the recommendations and documents from Sequence Variant Interpretation Working Group (https://www.clinicalgenome.org/working-groups/sequence-variant-interpretation/), and 2019 Guideline for Interpretation of Variant Pathogenicity recommended by Association for Clinical Genomic Science (ACGS), British Society for Genetic Medicine (https://www.acgs.uk.com/quality/best-practice-guidelines/), and classified into P, LP, VUS, likely benign and benign. P/LP as revealed by CMA and trio-WES were indicative of clinical significance.

### Statistical analysis

All data were entered into the Microsoft Excel 2021 (Microsoft Corporation; Redmond, WA, USA), and differences of proportions were tested for statistical significance with chi-square test. All statistical analyses were performed using the statistical software SPSS 25.0 (SPSS, Inc.; Chicago, IL, USA), and a *P* value of < 0.05 was considered statistically significant.

## Results

### Chromosomal abnormality detected by karyotyping analysis

Karyotyping analysis detected a 4.38%(17/388) proportion of chromosomal abnormality among the study participants, including 6 cases with chromosomal structural abnormality, 2 cases with trisomy 18, 2 cases with trisomy 21, 2 cases with triploidy, 2 cases with mosaic, 2 cases with 47, XXX syndrome and one case with Turner syndrome (Table 1).

**Table 1:** Abnormal karyotype analysis results among 17 cases with FGR

Case No.	Prenatal ultrasound findings	Karyotype	CMA results	Associated syndrome	CNVs classification	Pregnancy outcome
1	Absence of stomach bubble, axis offset, imbalance of the head-to-body ratio, asymmetrical FGR and abnormal limb posture	69,XXX	arr(X,1–22)× 3	69,XXX	P	TOP
2	FGR, fetal head circumference and humeral diameter smaller than two standard deviations below the mean	47,XXX	arr(X)× 3	47,XXX syndrome	P	TOP
3	FGR, biparietal diameter and head circumference smaller than three standard deviations below the mean Left cleft lip and palate, small stomach bubble, ventricular septal defect, small bilateral kidneys and right food inversion	46,XN, del(4)(p15.3)	arr[GRCh37]4p16.3p15.32(68346_15603607)x1	Wolf-Hirschhorn syndrome	P	TOP
4	FGR, plump anterior horns of fetal bilateral ventricles, flat face, lower lip retraction and small bilateral kidneys	46,XN, der(5)t(3:5)(p24.3;p14)	arr[GRCh37]3p26.3p24.3(61891_21383789)x3, 5p15.33p14.2(113576_23666841)x1	Cri-du-chat syndrome	LP	TOP
5	FGR, Fetal nasal bone dysplasia	46,XN, dup(1)(q21q22)	arr[GRCh37]1q21.1q21.2(143743406_143743447)x3,1q21.2q22(149752708_156221534)x3		P	TOP
6	FGR, fetal femoral and humeral lengths lower than three standard deviations below the mean, suspicion of bilateral subarachnoid cyst, ventricular septal defect, absence of venous catheter, and single umbilical artery	46,XN, del(13)(q12q13),	arr[GRCh37]13q12.2q13.3 (28780897_36857500)x1	Complex neurodevelopmental disorder	P	TOP
7	FGR, imbalance of the fetal head to the abdominal circumstance ratio, and fixed posture of bilateral hands	69,XXX, t(7;9)(q11.2;q34)	arr(X,1–22)× 3		P	TOP
8	FGR, fetal congenital heart disease	47,XN,+21	arr(21)× 3	Down syndrome	P	TOP
9	FGR, abnormal cardiac development, complete endocardial cushion defect, pulmonic stenosis, right aortic arch, persistent left superior cava, nasal bone dysplasia, echo enhancement of bilateral renal parenchyma and echogenic bowel	47,XN,+21	arr(21)× 3	Down syndrome	P	TOP
10	FGR, ventricular septal defect, and single umbilical artery	47,XN,+18	arr(18)× 3	Edwards syndrome	p	TOP
11	FGR, ventricular septal defect, large pulmonary artery to aorta ratio, small nasal bone and overlapping finger	47,XN,+18	arr(18)× 3	Edwards syndrome	P	TOP
12	FGR	45,XO	arr(X)× 1	Turner syndrome	P	TOP
13	FGR	47,XXX	arr(X)× 3	Super-female syndrome	P	TOP
14	FGR	47,XXX[62]/45,X[18]	Normal		P	TOP
15	FGR	47,XN,+psu idic(9)(q12)[39]/46,XN[11]	arr[GRCh37] 9p24.3q13 (208454_68216577)x4		P	TOP
16	FGR, widening of the left ventricle, ventricular septal defect, persistent left superior cava, tricuspid regurgitation and gallbladder enlargement	46,XN, add(16)(p13.3)	arr[GRCh37]16p13.3 (85880_536631)x1,17q24.2q25.3 (64966574_81041823)x3		P	TOP
17	FGR, thickened nuchal fold	46,XN, dup(12)(q14q23)	arr[GRCh37]12q14.2q23.1 (64877459_97710202)x3		P	TOP

*LP* Likely pathogenic, *P* Pathogenic, *TOP* Termination of pregnancy

### Chromosomal abnormality detected by CMA

In our study, case 14 was detected with sex-chromosome low-level mosaic (47,XXX[62]/45,X[18]) by karyotyping but not CMA, while case 53 was detected with low-level (10%) mosaicism of trisomy 16 and high-level (90%) mosaicism of ROH at p13.3p13.12 (44,807 − 13,885,927) by CMA but not karyotyping. Thus, the detection rate of chromosomal abnormality by CMA was estimated to be 13.4% (52/388, 36 + 16 = 52), including 35 cases with P CNVs, 4 cases with LP CNVs, 13 cases with VUS, and yielded a 9.28%(36/388) incremental detection rate over karyotyping analysis.

Karyotyping analysis in combination with CMA detected a 13.66% (53/388, 36 + 17 = 53) proportion of chromosomal structural abnormality. The additional chromosomal abnormalities detected by CMA included 16 cases with microdeletions, 11 cases with microduplications, 8 cases with regions of homozygosity (including one case with uniparental disomy (UPD)), and one case with chimera. The P/LP variants included one case with Wolf-Hirschhorn syndrome (case 45), one case with Xq28 recurrent region (case 24), one case with Xq28 microduplication syndrome (case 29), one case with 16p13.3 microdeletion syndrome (case 23), 2 cases with 22q11.21 microduplication syndrome (cases 18 and 14), 2 cases with 22q11.21 microdeletion syndrome (cases 28 and 49), one case with 22q13.2q13.33 microdeletion syndrome (case 33), and one case with UPD (case 48). The microdeletions ranged from 79.6 kb to 15.53 Mb in length, and microduplications ranged from 0.25 Mb to 6.47 Mb in length (Table 2).

**Table 2:** LP/P and VUS CNVs detected by CMA among 36 cases with FGR negative for karyotype analysis

Case No.	Prenatal ultrasound findings	CMA results	Aberration/size	Inheritance	Associated syndrome	CNVs classification	Pregnancy outcome
18	FGR	arr[GRCh37]22q11.21 (18916842_21464764)x3	dup/2.18 Mb	De novo	22q11.2 recurrent (DGS/VCFS) region (proximal, A-D)(key geneTBX1), 22q11 duplication syndrome	P	TOP
19	FGR, head circumference smaller than 2 standard deviations below the mean	arr[GRCh37] 1p35.2p35.1 (31005858_33099109)x1	del/2.10 Mb	De novo		VUS	TOP
20	FGR	arr[GRCh37] 16p13.3p12.3(94807_ 17270944)× 2 hmz	LOH/17.18 Mb	NA		VUS	TOP
21	FGR, heart enlargement, pleural and abdominal effusion	arr[GRCh37] 16p13.11 (15481747_ 16282869)x3	dup/0.80 Mb	De novo	16p13.11 recurrent microduplication (The penetrance of the gene expression is 5%−10%)	VUS	TOP
22	FGR	arr[GRCh37]4p13p12 (4423136_45803999) x4, 15q26.2q26.3(97218700_102429040)x1	4dup/1.59 Mb, 15del/5,21 Mb	4Pat,15mat	Growth delay which is associated with insulin-like growth factor 1 resistance	VUS/LP	TOP
23	FGR	arr[GRCh37]16p13.3(3750606_3827552)x1	del/0.08 Mb	De novo	Involving the CREBBP gene, the clinical presentation is delayed intrauterine growth, microcephaly. Since the missing fragment was 79.6 kb, which was lower than the detection range, Trio-WES was used to verify the heterozygosity deletion of exons 11,12,22,23,31 of CREBBP gene, indicating a new mutation.	P	TOP
24	FGR, single umbilical artery	arr[GRCh37]Xq28 (154120632_154564050)x2	dup/0.44 Mb	Mat	Xq28 recurrent region, which contains the RAB39B gene, can be characterized by developmental delay and intellectual disability. Female carrier, male disease, this fetus is male fetus.	P	TOP
25	FGR	arr[GRCh37]6p25.3q27 (203877_170896644)x2hmz	LOH/UPD(mat)	De novo	The probability of suffering from recessive genetic diseases increases.	VUS	TOP
26	FGR	arr[GRCh37]16p13.11(14929070_16278132)x3	dup/1.35 Mb	De novo	16p13.11recurrent microduplication (The penetrance of the gene expression is 5–10%)	VUS	TOP
27	FGR	arr[GRCh37]1q41q42.11(222693029_224184261)x1	del/1.48 Mb	NA		VUS	Healthy full-term infant
28	FGR, head circumference and humeral diameter less than 2 standard deviations below the mean, fetal nasal bone hypoplasia	arr[GRCh37]22q11.21.q11.22(21800471_22962962)x1	del/1.17 Mb	De novo	22q11.2 distal deletion syndrome	P	TOP
29	FGR	arr[GRCh37]Xq28(153621056_153868486)x2	dup/0.25 Mb	Mat	Xq28 microduplication syndrome (containing GDI1 gene)	P	TOP
30	FGR	arr[GRCh37]21q21.1q22.13(22819791_39257377)x2hmz	LOH/16.44 Mb	NA		VUS	Healthy full-term infant
31	FGR, fetal head circumference smaller than 3 standard deviations below the mean, thickening of the anterior soft tissue of the fetal nose	arr[GRCh37] 6p21.31p21.2(36168300_39466613)x1	del/3.3 Mb	De novo	The main clinical features are congenital progressive microcephaly and postnatal psychomotor dysplasia or absence accompanied by severe intellectual impairment.	VUS	TOP
32	FGR, ventricular septal defect, high-intensity echo of left ventricles	arr[GRCh37] 2p16.1(56788428_58572494)x4	dup/1.78 Mb	NA	The symptoms are intrauterine growth retardation, congenital heart defects, renal dysplasia, esophageal atresia, and skeletal malformations.	VUS	TOP
33	FGR, hydronephrosis of left kidney, dilation of upper segment of the left ureter, slight echo enhancement of renal parenchyma, accessory renal arteries of bilateral kidneys, and slight echo enhancement of local intestine	arr[GRCh37] 22q13.2q13.33(42640606_ 51197766)x1	del/8.5 Mb	NA		P	TOP
34	FGR, advanced age	arr[GRCh37] 16p11.2(29591327_ 30190029)x3	dup/599Kb	NA	The clinical phenotype of patients is heterogeneous, with abnormalities such as developmental delay, autism spectrum disorder, cognitive impairment, behavioral problems (including attention deficit and hyperactivity disorder) and microcephaly, and some patients may have no obvious clinical abnormalities.	P	TOP
35	FGR, advanced age	arr[GRCh37] 15q11.2q13.1(22770422_28526905)x3	dup/5.75 Mb	Mat	The repeats in this region have a parental effect, with maternal repeats are usually associated with abnormal phenotypes and most of them phenotypes normal.	P	TOP
36	FGR, exophthalmos, partial echogenic bowel (grade II), suspicion of Crouzon syndrome	arr[GRCh37] 5q21.2q31.3(103612677_140570644)x2 hmz	LOH/36.9 Mb	NA	This ROH includes 22 genes associated with autosomal recessive genetic diseases, such as SLC25A46 (610826) and HSD17B4 (601860), increasing the risk of autosomal recessive genetic disorders due to homozygous mutations.	VUS	TOP
37	FGR	arr[GRCh37] 12p13.2p12.1(10143600_ 22685434)x2 hmz	LOH/12.5 Mb	NA	LOH contains 9 genes including CLEC7A (606264), GUCY2C (601330), MGP (154870) and other genes associated with autosomal recessive disorders. At present, there are no clear imprinted genes in this region, but the risk of autosomal recessive diseases is increased.	VUS	TOP
38	FGR, portal venous trafficking, small bilateral kidneys, nasal bone dysplasia, advanced age	arr[GRCh37] 4p16.3p15.1(68345_35252743)x1	del/35 Mb	NA		P	TOP
39	FGR, permanent left superior vena cava, ventricular septal defect, polyhydramnios, left crossed ectopic kidney	seq[GRCh37]6q21(106534427_108831555)x1	del/2.30 Mb	De novo		VUS	TOP
40	FGR, abnormal umbilical artery blood flow spectrum and enhanced local echo in the intestine	seq[GRCh37]22q11.23(23915451 _24922115)x3	dup/1.01 Mb	Mat		VUS	Intrauterine fetal deaths at gestational ages of 29 weeks
41	FGR, polyhydramnios	arr[GRCh37] 15q14q21.3(35077111_54347324)x2 hmz	LOH/19.27 Mb	NA		P	TOP
42	FGR	arr[GRCh37] 7q11.23(72723370_74143240)x1	del/1.4 Mb	De novo		P	TOP
43	FGR, permanent left superior vena cava, narrow inner diameter of the aortic arch, and mild echo enhancement of bilateral renal parenchymal	arr[GRCh37] 2p25.3p11.2(50813_87053152)x2hmz, 2q11.1q37.3 (95550957_ 242773583)x2 hmz	LOH/87 Mb, LOH/147.22 Mb	UPD(mat)		LP	TOP
44	FGR, nuchal fold thickening, fetal intrahepatic portosystemic venous shunt, intraabdominal tumor dilation of the umbilical vein, enlarged cardiothoracic ratio, and mild tricuspid regurgitation	arr[GRCh37] 22q11.21 (18648855_ 21459713)x3	dup/2.8 Mb	Mat		P	TOP
45	FGR, fetal pulmonary artery stenosis	arr[GRCh37] 4p16.3p16.1(68345_6608624)x1	del/6.5 Mb	NA	Wolf-Hirschhorn syndrome	P	TOP
46	FGR, ventricular septal defect, pulmonary valve stenosis complicated by insufficiency and nasal bone dysplasia	arr[GRCh37] 15q24.1q24.2(72965465_75567135)x1	del/2.6 Mb	De novo	15q24 mircrodeletetion syndrome	P	TOP
47	FGR, single umbilical artery, less amniotic fluid, thickening of placenta	arr[GRCh37] 8q11.23q12.1(54456444_59599862)x1	del/5.1 Mb	NA		LP	TOP
48	FGR, mild tricuspid regurgitation, inverted α wave on venous catheters, and echogenic bowel	arr[GRCh37] 6p25.3q27(203877_170896644) x2 hmz	LOH/170.38 Mb	UPD		P	TOP
49	FGR, ventricular septal defect	arr[GRCh37] 22q11.21(18,648,855_21800471)×1	del/3.1 Mb	NA	22q11.2 distal deletion syndrome	P	TOP
50	FGR, incomplete ventricular septal pulmonary atresia, severe tricuspid regurgitation	arr[GRCh37]17p11.2(16615982_18922171)×3	del/2.1 Mb	Mat	Potocki-Lupski syndrome	P	TOP
51	FGR, echogenic bowel	arr[GRCh37]10q11.22q11.23(46252072_51903756) ×1	del/5.6 Mb	De novo		P	TOP
52	FGR, femur and humerus located below 2 standard deviations below the mean	seq[GRCh37]Xp22.33(200,853_ 605,604)x1	del/0.40 Mb	De novo	Leri-Weill dyschondrostosis syndrome	P	TOP
53	FGR, small fetal aorta and small left ventricle	arr(16)×3[0.1], 16p13p13.3p13.12(44807_13885927)ROH[0.9]	ROH/13.83 Mb	De novo	Low-level (10%) mosaicism of trisomy 16. The clinical phenotype of trisomy is variable, which can be manifested as growth retardation, congenital heart defects, lung hypoplasia, limb abnormalities, and hypospadias.	P	TOP

*Mat* Maternal, *Pat* Paternal, *NA* Not available, *VUS* Uncertain clinical significance, *LP* Likely pathogenic, *P* Pathogenic, *TOP* Termination of pregnancy

### Gene abnormality detected by trio-WES

26 pregnant women applied for additional trio-WES with negative karyotyping and CMA after genetic counseling. Trio-WES revealed P/LP variants among 8 (30.77%) fetuses, and 11 genes were identified, including *IGF1R* (case 54), *COL9A3* (case 57), *FGA* (case 57), *EVC2* (case 58), *NEK8* (case 58), *SPINK1* (case 60), *RP1* (case 62), *SCN5A* (case 56), *GJB2* (case 63), *GNAS* (case 64) and *FGD1* (case 68). Among them, the growth- and development-related genes included *IGF1R*, *GNAS*, *COL9A3* and *FGD1.* VUS was found among 7 (26.92%) fetuses and 7 genes were identified, including *FLNB* (case 55), *TRPS1* (case 56), *PORCN* (case 59), *EVC* (case 61), *OTX2* (case 65), *GH1* (case 66) and *COC4A1* (case 67), among which the growth- and development-related genes included *FLNB*, *EVC*, *OTX2* and *GH1*. (Table 3).

**Table 3:** LP/P and VUS gene mutation detected by trio-WES among 15 cases with FGR negative for karyotyping and CMA

Case No.	Maternal age (weeks)	Gestational age at trio-WES (weeks)	Prenatal ultrasound findings	Gene	Chromosome coordinates/bases/amino acids (GRCh37/hg19)	Mutation type	Gene subregion	Type of variant	Inheritance mode	Associated syndrome	Gene mutation classification	Outcomes
54	28	31w + 3d	FGR (*P* < 3%)	IGIF1R (NM_000875.4)	Chr15:99465356–99,467,933.ex.11-13del/p.?	HET	Exons 11 to 13	De novo	AD, AR	Insulin-like growth factor I resistance	LP	TOP
55	34	27w + 6d	FGR, fetal femur and humerus located below 2 standard deviation below the mean	FLNB (NM_001457.3)	Chr3:58088020/c.1436 C > T/P.Thr479IIe	HET	Exon9	Mat	AD, AR	Spondylocarpotarsal synostosis syndrome, Larsen syndrome	VUS	TOP
56	32	25w + 5d	FGR, fetal femur and humerus located 3 standard deviations below the mean	TRPS1 (NM_014112.4)	Chr8:116632278/c.47G > A/p.Arg16Gln	HET	E:3/7	De novo	AD	Trichorhinophalangeal syndrome type I (OMIM: 190350) and type III (OMIM: 190351)	VUS	Full-term small-for-gestational-age infant
57	29	22w	FGR	COL9A3 (NM_001853.3); FGA (NM_021871.3)	Chr20:61470070/c.1821dupA/p.G608RFs*8; Chr4:155506882/c.1690-1699dupCACCCTGGGA/p.lle567fs	HET; HET	E:31/32; E:5/5	Pat; mat	AD; AR	Multiple epiphyseal dysplasia type 3 with or without myopathy (OMIM: 600969), familial visceral amyloidosis (OMIM: 105200)	LP	TOP
58	30	22w + 4d	FGR, fetal nasal bone dysplasia	EVC2 (NM_147127.5); NEK8 (NM_178170.3)	Chr45633572/c.1655_1658del /p.Gly552Aspfs*2; Chr1727064747/c.882del /p.Cys295Alsfs*9	HET; HET	Exon11; Exon6	Pat; mat	AD; AR	Weyers manillary hypoplasia; polycystic kidney disease type 8	P; LP	TOP
59	24	24w + 2d	FGR, smaller kidneys; fetal microphthalmos	PORCN (NM_203475.3)	ChrX:48,372,996/c.929 C > A/p.Ser310Tyr	HET	Exon10	Mat	XLD	Focal dermal hypoplasia (Goltz syndrome; OMIM: 3 00651), short stature, abnormal eye development (this is a male fetus)	VUS	TOP
60	37	26w + 5d	FGR, left polycystic dysplasia kidney	SPINK1 (NM_003122.4)	Chr5:147207583/c.194 + 2 T > C	HET	1:4/4	Mat	AD, AR	Fibrocalculous pancreatic diabetes (OMIM: 608189); Tropical calcific pancreatitis (OMIM: 608189); hereditary pancreatitis (OMIM: 167800)	P	Healthy full-term infant
61	26	26w + 5d	FGR, Polydactyly of the fetal hands, fetal femur and humerus located below 2 standard deviations below the mean	EVC (NM_153717.3)	Chr4:5731118/c.384 + 1G > T	HET	1:3/20	Mat	AD, AR	Ellis-van Creveld syndrome (OMIM: 225500), Weyers manillary hypoplasia) (OMIM: 193530)	VUS	TOP
62	34	26w + 5d	Fetal biparietal diameter below 2 standard deviations below the mean, FGR	RP1 (NM_006269.2)	Chr8:55542239/c.5797 C > T/ p.Arg1933	HET	E:4/4	Mat	AD	Retinitis pigmentosa type 1 (OMIM: 180100),	P	Healthy full-term infant
63	25	24w + 5d	FGR, central brain cyst, suspicion of arachnoid cyst	SCN5A (NM_001099404.2); GJB2 (NM_004004.6)	Chr3:38622757/ c.2893 C > T/p.Arg965Cys; Chr13:20763612/c.109G > A/p.Val37Ile	HET; HOM	E:17/28 E:2/2	Mat; parents	AD; AR	Brugada syndrome type 1; long QT syndrome type 3; Autosomal recessive deafness type IA (P)	LP/P	TOP
64	33	25w + 2d	FGR, oligohydramnios	GNAS (NM_000516.5)	Chr20:57480498/c.493 C > T/p.Arg165Cys	HET	E:6/13	De novo	AD	Pseudohypothyroidism, progressive bone development (OMIM: 103580; 603233); progressive osteodystrophy (OMIM:166350); delayed growth and intellectual development	P	TOP
65	24	30w + 5d	FGR	OTX2 (NM_172337.3)	Chr14:57270949/c.206 A > G/p.Glu69Gly	HET	Exon2	De novo	AD	Syndromic microphthalmia type 5	VUS	TOP
66	42	25w	FGR, advanced age	GH1 (NM_000515.5)	Chr17:61995162/c.414 C > A/p.Asp138Glu	HET	Exon4	De novo	AR	Isolated growth hormone deficiency, type II	VUS	TOP
67	31	23w + 3d	FGR, absence or dysplasia of right kidney, large left kidney	COL4A1 (NM_001845.6)	Chr13:110813696/c.4483 C > T/P.Arg1495Cys	HET	Exon49	De novo	AD	Hereditary angiopathy with nephropathy, aneurysm and muscle spasm and leukoencephalopathy	VUS	TOP
68	31	25w + 5d	FGR, fetal femur and humerus located below 2 standards deviation below the mean, persistent left superior vena cava	FGD1 (NM_OO4463.3)	ChrX:54,497,015/c.659 + 1G > A	HEMI	1:3/17	Mat	XLR	Aarskog-Scott syndrome, X-linked mental retardation syndrome type 16 (this is a male fetus)	LP	Premature birth

*HOM* Homozygous variant, *HET* Heterozygous variants, *HEMI* Hemizygous, *Mat* Maternal, *Pat* Paternal, *AD* Autosomal dominant, *AR* Autosomal recessive; XLD, X-linked dominant; XLR, X-linked recessive; TOP, termination of pregnancy

### Comparison of the efficiency of karyotyping, CMA and trio-WES alone and in combinations for detection of chromosomal and gene abnormality.

There was a significant difference in the detection of chromosomal and gene abnormality among the participants by karyotyping (4.38%), CMA (13.4%) and trio-WES (57.69%) (*P* < 0.001).

Karyotyping analysis combined with CMA detected the highest chromosomal abnormality rate (48.48%, 16/33)in the FGR complicated by ultrasound structural abnormality group, followed by the FGR complicated by multiple ultrasound soft markers group (26.92%, 14/52), the FGR complicated by a single ultrasound soft marker group (7.89%, 6/76) and the isolated FGR group (7.48%, 17/227)(*P* < 0.001); however, no significant difference was seen between the FGR complicated by a single ultrasound soft marker group and the isolated FGR group (*P* > 0.05).

Trio-WES detected the highest genetic mutation rate in the FGR complicated by ultrasound structural abnormality group (66.67%, 4/6), followed by the FGR complicated by multiple ultrasound soft markers group (80%, 4/5), the FGR complicated by a single ultrasound soft marker group (75%, 3/4) and the isolated FGR group (36.36%, 4/11) (*P* > 0.05) (Tables 4, 5 and 6).

**Table 4:** Detection of chromosomal and gene abnormality with karyotyping, CMA and Trio-WES in pregnancies with FGR

Method	No. tested	No. of positive results	Positive rate (%)
Karyotyping	388	17	4.38
CMA	388	52	13.4
Karyotyping + CMA	388	53	13.66
Trio-WES	26	15	57.69

There is a significant difference in the detection of chromosomal and gene abnormality using the four tests (χ^2^ = 76.912, *P* < 0.001)

Karyotyping vs. CMA, χ^2^ = 19.486, *P* < 0.001; karyotyping vs. karyotyping + CMA, χ^2^ = 20.350, *P* < 0.001; karyotyping vs. Trio-WES, χ^2^ = 89.770, *P* < 0.001; CMA vs. Trio-WES, χ^2^ = 32.049, *P* < 0.001; karyotyping + CMA vs. Trio-WES, χ^2^ = 31.284, *P* < 0.001

**Table 5:** Detection of chromosomal abnormality with karyotyping alone and in combination with CMA in pregnancies with FGR

Group	Cases	Abnormality detected by karyotyping + CMA (%, *n*)	Abnormality detected by karyotyping (%, *n*)	Abnormality detected by CMA with negative karyotyping (%, *n*)	
				VUS	LP/*P*
Isolated FGR (1)	227	7.48 (17)	1.76 (4)	3.08 (7)	2.64 (6)
FGR complicated by a single ultrasound soft marker (2)	76	7.89 (6)	2.63 (2)	2.63 (2)	2.63 (2)
FGR complicated by multiple ultrasound soft markers (3)	52	26.72 (14)	7.69 (4)	5.76 (3)	13.46 (7)
FGR complicated by ultrasound structural abnormality (4)	33	48.48 (16)	21.21 (7)	6.06 (2)	21.21 (7)

A significant difference is found in the detection of chromosomal abnormality using karyotyping in combinations with CMA among the four groups (χ^2^ = 51.161, *P* < 0.001). Group 1 vs. Group 2, χ^2^ = 0.013, *P* > 0.05; Group 1 vs. Group 3, χ^2^ = 16.179, *P* < 0.001; Group 1 vs. Group 4, χ^2^ = 43.697, *P* < 0.001; Group 2 vs. Group 3, χ^2^ = 8.480, *P* < 0.05; Group 2 vs. Group 4, χ^2^ = 23.532, *P* < 0.001; Group 3 vs. Group 4, χ^2^ = 4.110, *P* < 0.05

A significant difference is found in the detection of chromosomal abnormality using karyotyping among the four groups (χ^2^ = 19.261, *P* < 0.001). Group 1 vs. Group 2, adjusted χ^2^ = 0, *P* > 0.05; Group 1 vs. Group 3, adjusted χ^2^ = 3.425, *P* > 0.05; Group 1 vs. Group 4, χ^2^ = 22.314, *P* < 0.001; Group 2 vs. Group 3, adjusted χ^2^ = 0.818, *P* > 0.05; Group 2 vs. Group 4, adjusted χ^2^ = 8.177, *P* < 0.05; Group 3 vs. Group 4, adjusted χ^2^ = 2.185, *P* > 0.05

A significant difference is found in the detection of LP/P CNVs using CMA in pregnancies with normal karyotypes among the four groups (χ^2^ = 19.803, *P* < 0.001). Group 1 vs. Group 2, adjusted χ^2^ = 0, *P* > 0.05; Group 1 vs. Group 3, adjusted χ^2^ = 9.767, *P* < 0.05; Group 1 vs. Group 4, χ^2^ = 14.905, *P* < 0.001; Group 2 vs. Group 3, adjusted χ^2^ = 4.401, *P* > 0.05; Group 2 vs. Group 4, adjusted χ^2^ = 6.945, *P* < 0.05; Group 3 vs. Group 4, adjusted χ^2^ = 0.350, *P* > 0.05

**Table 6:** Detection of gene abnormality among 26 pregnant women by Trio-WES

Group	Case	Negative cases	Positive rate(%)	Rate of gene mutation(%)		
				VUS	LP/*P*	
Isolated FGR	11	7	36.36 (4/11)	18.18 (2/11)	18.18 (2/11)	
FGR complicated by a single ultrasound soft marker	4	1	75.00 (3/4)	0 (0/4)	75 (3/4)	
FGR complicated by multiple ultrasound soft markers	5	1	80.00 (4/5)	40 (2/5)	40(2/5)	
FGR complicated by ultrasound structural abnormality	6	2	66.67 (4/6)	50 (3/6)	16.67 (1/6)	
Total	26	11	57.69 (11/26)	26.92 (7/26)	30.77 (8/26)	

No significant difference is seen in the detection of gene abnormality by Trio-WES among the four groups (χ^2^ = 3.504, *P* > 0.05)

### Pregnancy outcomes

Of 320 pregnancies with FGR that were negative for genetic testing, there were 239 healthy full-term infants, 17 full-term small-for-gestational-age infants, 12 preterm births, 16 induced labors, 2 abortions, 2 intrauterine fetal deaths after 28 weeks of gestational age, 4 neonatal deaths within 4 months of life, and 28 pregnancies lost to follow-up. Seventeen pregnancies with abnormal karyotypes were all induced of labors, and of the 36 pregnancies tested negative by karyotyping and positive by CMA, there were 23 pregnancies with P/LP CNVs that were all induced of labors. In addition, of the 8 pregnancies detected with P/LP variants by trio-WES, the pregnancy outcomes included induction of labors in 5 pregnancies, 2 healthy full-term infants, and one preterm birth. During genetic counseling of VUS detected by CMA and trio-WES, VUS is not recommended as an indicator of termination of pregnancy, and the pregnant woman and her spouse are recommended to undergo CMA. In this study, unfortunately, there were still 16 couples that decided to terminate the pregnancy due to multiple ultrasound soft markers, fetal structural abnormalities or progressive FGR.

(Table 7).

**Table 7:** Follow-up outcomes of 388 pregnancies

Follow-up outcome		Healthy full-term infant	Full-term small-for-gestational-age infant	Preterm birth	Induced labor	Abortion	Intrauterine fetal deaths after 28 weeks of gestational age	Neonatal deaths within 4 months of life	Loss to follow-up	Total
Negative genetic tests		239	17	12	16	2	2	4	28	320
Positive karyotyping		0	0	0	17	0	0	0	0	17
Positive CMA and negative karyotyping	Likely pathogenic/pathogenic	0	0	0	23	0	0	0	0	23
	VUS	2	0	0	10	0	1	0	0	13
Positive Trio-WES	Likely pathogenic/pathogenic	2	0	1	5	0	0	0	0	8
	VUS	1	0	0	6	0	0	0	0	7
Total		244	17	13	77	2	3	4	28	388

## Discussion

In the current study, seventeen (4.38%) cases of chromosomal abnormalities were detected by cytogenetic karyotyping, including trisomy 18, trisomy 21, sex chromosome abnormality and triploidy. Trisomy 18 and trisomy 21 were the most common chromosomal abnormalities, which is similar to previous reports [11]. In addition, karyotyping detected the highest proportion of chromosomal abnormality in the FGR complicated by ultrasound structural abnormality group (21.21%), followed by the other three groups. (*P* < 0.05), which is similar to our previous findings [8]. Currently, cytogenetic karyotyping remains the gold standard for cytogenetic diagnosis, demonstrating exceptional efficacy in detection of numerical and structural chromosome abnormalities [18]. In this study, karyotyping detected sex-chromosome low-proportion chimerism (47,XXX[62]/45,X [18]) in case 14, which was not identified by CMA. In presence of particular chimerism levels, CMA fails to detect the detailed components of the sex chromosome, if no remarkable changes occur in chromosome dosage. Therefore, karyotyping remains an indispensable assay for prenatal genetic diagnosis [19].

In the present study, among pregnancies with normal karyotype, an incremental yield of 9.28% were revealed by CMA, indicating the higher detection of chromosomal abnormality in pregnancies with FGR by CMA than karyotyping [7, 20]. The highest detection rates by CMA is in the FGR complicated by ultrasound structural abnormality group (21.21%), which was lower than reported in Israel (29.4%) [21], but higher than the pooled prevalence from January 2009 to November 2016 [7] and the report in France [22]. This may be attributed to FGR complicated by different types of ultrasound structural abnormalities. In this study, we detected 2 cases with 22q11.21 microdeletion and microduplication syndromes complicated by structural heart abnormality, which was similar to previous studies reporting that 22q11.21 microdeletion syndrome is likely to be complicated by FGR and cardiac abnormality [23, 24]. 4p16.3 microdeletion syndrome, which is Linked with Wolf-Hirschhorn syndrome, was detected in cases 3, 38 and 45. Its major intrauterine clinical manifestations included severe growth and developmental retardation at middle and late pregnancies [25], which may be complicated by congenital heart disease, urogenital system and facial disorders [26]. Xq28 microduplication syndrome was detected at the Xq region in cases 24 and 29, containing *RAB39B* and *GDI1* gene, and this may cause intellectual disability, growth and developmental retardation and dysgenesis of the corpus callosum [27, 28]. Case 52 was detected with a 0.40 Mb deletion at Xp22.33, which involved *SHOX* gene, and defect of the *SHOX* gene may cause Leri-Weill dyschondrosteosis syndrome, which is characterized by mild non-specific short stature and severe achondroplasia [29, 30]. In this study, Case 53 showed low-level (10%) mosaicism of trisomy 16 and high-level (90%) mosaicism of ROH at p13.3p13.12 (44,807 − 13,885,927) by CMA but not karyotyping, which may be explained by the possibility of culture selection of the normal cell Line. FISH is recommended to test the reliability of chimeras, however, the pregnant women refused and chose to terminate the pregnancy. CMA analysis of samples from both maternal and fetal surfaces of placenta revealed trisomy 16. This may be explained by confined placental mosaicism (CPM), which indicates that chromosomal abnormality is restricted in uterine, but fetal chromosome is normal. These mosaic embryos are error consequences of zygotes, namely chromosomal segregation error during the first mitosis. Most cells in fetuses self-rescue to discard redundant trisomy, however, a few mosaic trisomy remains [31]. It is reported that CPM is diagnosed with non-invasive DNA testing for capturing abnormal signals, followed by definite diagnosis with invasive prenatal diagnosis [32]. In this study, case 53 was diagnosed with trisomy 16 by non-invasive DNA testing of peripheral blood due to advanced age at gestational age of 13 weeks. The prevalence of prenatal FGR is reported to be 71.7% among patients with CPM [31], and the highest incidence of CPM is detected on chromosome 16 [33, 34].

Our findings showed that karyotyping combined with CMA increased the detection of chromosomal abnormality in pregnancies with FGR, however, the etiology of FGR remained unknown in 86.34% of pregnancies. Trio-WES detected an incremental yield of 57.69% (15/26) among 26 cases with normal karyotype and CMA results, including 8 P/LP and 7 VUS variants. Case 54 was detected with severe isolated FGR after 28 weeks of gestational age, and Trio-WES revealed a novel IGF1R gene mutation. Insulin-like growth factor-1 (IGF-1) has been found to stimulate important cell processes, including proliferation, division and metabolism [35]. Individuals carrying IGF1R gene mutation are found to manifest as intrauterine growth retardation, short stature and microcephaly after birth [36]. Case 64 was identified with GNAS gene mutation which may cause pseudohypoparathyroidism manifested as hypocalcemia, short stature and ectopic ossification [37, 38]. Case 57 was detected with COL9A3 gene mutation that is associated with multiple epiphyseal dysplasia (OMIM: 132400) [39]characterized by moderate or mild short stature and early-onset osteoarthritis [40]. COL9A3 gene is autosomal dominant and identified from the father, while the father has no abnormal clinical phenotypes. The aforementioned cases all decided to terminate the pregnancy. Case 68 was detected with FGD1 gene mutation and maternal X-linked recessive(XLR)inheritance. The mutation of the FGD1 gene may cause ocular hypertelorism, short stature, and genital abnormalities [41, 42]. Pedigree analysis revealed that the FGD1 gene were derived from the maternal grandfather, which had a body height of 160 cm, ocular hypertelorism and short fingers while the mother had no clinical phenotype. A healthy premature infant was delivered in case 68. Findings from cases 57 and 68 highlight the fact that pathogenic variants may have variable expressivity and incomplete penetrance, and highlight the importance of acquiring the complete medical records and genetic testing of other family members. Of all pregnancies with VUS, there were four pregnancies with VUS that were associated with the FGR phenotype, involving FLNB (AD, mat), PORCN (XLD, mat), EVC (AR, AD, mat), and GH1 genes (AD, de novo). Nevertheless, all pregnant women decided to terminate the pregnancy.

In our study, Case 23 was identified with a 0.08 Mb heterozygous deficiency at 16p13.3 using CMA at 27 weeks of gestation age, which involved the *CREBBP* gene. Since the deletion fragment was 79.6 kb, which was lower than the detection range, trio-WES analysis of exons 11, 12, 22, 23 and 31 on the *CREBBP* gene confirmed heterozygous deficiency and de novo mutation. Mutation of the *CREBBP* gene may cause Rubinstein-Taybi syndrome, which is characterized by delayed growth and motor development, abnormal gross motor function and mild to severe intellectual disability [43]. The pregnancy was terminated following clinical genetic counseling at 32 weeks of gestational age. This case suffered from a single-gene disorder, and if trio-WES was directly performed, this may shorten the gestational age at termination of pregnancy, which may alleviate the injuries to pregnant women.

WES has been identified as a first-tier diagnostic test for rare genetic disorders [44]. Emms and colleagues [45] reported that WES gained an 18.7% incremental yield for diagnosis of congenital abnormalities over CMA, and a recent meta-analysis showed a 31% additional diagnostic yield of WES over CMA for prenatal diagnosis of non-immune hydrops fetalis [46]. In addition, a recent meta-analysis examining the diagnostic yield of WES in 146 fetuses with FGR showed an incremental yield of 12%(17/146) over negative CMA and karyotyping [47]. In this study, an additional trio-WES analysis was performed in 26 pregnancies with FGR that were negative for CMA and karyotyping, resulting in a 30.77% (8/26) incremental yield for detection of LP/P variants, which is similar to a previous study reporting the incremental yield in isolated FGR with short long bones(33.3%) [48]. The heterozygous mutations in IGF1R, *GNAS*, *COL9A3* and *FGD1* genes may be directly associated with the phenotype of FGR. These findings demonstrate that IGF1R, *GNAS*, *COL9A3* and *FGD1* may be candidate genes responsible for FGR.

In the current study, we followed up the pregnancy outcomes in 388 pregnancies with FGR, and found a live birth rate of 70.62% (274/388) and a perinatal mortality rate of 1.8% (7/388), which is higher than the average perinatal mortality in China during the latest five years (0.393–0.414%) [49, 50]. Our data demonstrate that FGR may increase the risk of perinatal mortality. In this study, trio-WES detected VUS and LP/P gene mutations in 15 out of 26 pregnancies. Cases 60, 62 and 68 were detected with LP/P variants, however, their ultrasound findings were inconsistent with major clinical phenotypes, and inherited from mothers. The pregnant women decided to continue the pregnancy and healthy infants were finally delivered. Our finding demonstrate that detection of LP/P variants using trio-WES may not be indicative of poor fetal prognosis, and the termination of pregnancy is required to be decided based on comprehensive assessments of clinical phenotypes, pathogenicity, source of genetic variations and penetrance and clinical counseling.

This study has some Limitations. First, pregnant women identified with VUS tended to decide to terminate the pregnancy, and we failed to follow up the fetal prognosis. Second, only 26 pregnancies underwent trio-WES, and the real value of trio-WES for diagnosis of chromosomal abnormality cannot be precisely evaluated in pregnancies with FGR. Third, Should we inform the mother about the secondary findings of P/LP variants that are not associated with the major clinical phenotypes of FGR but occasionally identified by trio-WES? Since these accidental findings may affect the whole family. Fourth, prenatal WES is different from postnatal WES in depth and width. Prenatal WES suffers from a problem of underdiagnosis due to limitations of intrauterine phenotypes, which results in more difficulties and challenges for prenatal genetic counseling. Fifth, interpretation of VUS reports is a clinical challenge, and inappropriate interpretation may increase maternal anxiety and lead to adverse pregnancy outcomes. With increasing understanding of human genome and the accumulation of available data, the proportion of VUS may gradually decrease.

## Conclusions

In summary, karyotyping combined with CMA remains the first-tier tool for prenatal genetic diagnosis of pregnancies with FGR, and additional trio-WES may increase the detection and reduce the underdiagnosis of gene abnormality. The highest prevalence of chromosomal abnormality is detected in fetuses with FGR complicated by ultrasound structural abnormality, followed by those with FGR complicated by multiple ultrasound soft markers, and the common microdeletion and microduplication syndromes detected in pregnancies with FGR include 4p16.3.3 microdeletion syndrome, 1q21.2 microduplication syndrome, 16p13.3 microdeletion syndrome and 22q11.21 microduplication syndrome. In addition, IGF1R, *GNAS*, *COL9A3* and *FGD1* may be candidate genes causing FGR.

## Data Availability

All data presented in this study are available upon request by contact with the corresponding author.

## Abbreviations

FGR: Fetal growth restriction
CMA: Chromosomal microarray analysis
WES: Whole exome sequencing
trio-WES: Trio-based whole exome sequencing
ACOG: American College of Obstetricians and Gynecologists
EDTA: Ethylenediaminetetraacetic acid
CNV: Copy number variation
DGV: Database of Genomic Variants
OMIM: Online Mendelian Inheritance in Man
VUS: Uncertain clinical significance
ACMG: American College of Medical Genetics and Genomics
MLPA: Multiplex ligation-dependent probe amplification
FISH: Fluorescence in situ hybridization
SNP: Single nucleotide polymorphism
qPCR: Quantitative fluorescent polymerase chain reaction
LOH: Loss of heterozygosity (LOH)
AOH: Absence of heterozygosity
ACGS: Association for Clinical Genomic Science
UPD: Uniparental disomy
